# Role of angiotensin-converting enzyme in myeloid cell immune responses

**DOI:** 10.1186/s11658-020-00225-w

**Published:** 2020-05-25

**Authors:** Duo-Yao Cao, Suguru Saito, Luciana C. Veiras, Derick Okwan-Duodu, Ellen A. Bernstein, Jorge F. Giani, Kenneth E. Bernstein, Zakir Khan

**Affiliations:** 1 Department of Biomedical Sciences, Cedars-Sinai Medical Center, Los Angeles, CA 90048 USA; 2 Department of Pathology and Laboratory Medicine, Cedars-Sinai Medical Center, Davis Res. Bldg., Rm. 2014, 8700 Beverly Blvd, Los Angeles, CA 90048 USA

**Keywords:** Angiotensin-converting enzyme, Myeloid cells, Immune response, Neutrophils, Macrophages, Dendritic cells, Hematopoiesis, Methicillin-resistant *Staphylococcus aureus* (MRSA), Melanoma, MHC class I antigen presentation

## Abstract

Angiotensin-converting enzyme (ACE), a dicarboxypeptidase, plays a major role in the regulation of blood pressure by cleaving angiotensin I into angiotensin II (Ang II), a potent vasoconstrictor. Because of its wide substrate specificity and tissue distribution, ACE affects many diverse biological processes. In inflammatory diseases, including granuloma, atherosclerosis, chronic kidney disease and bacterial infection, ACE expression gets upregulated in immune cells, especially in myeloid cells. With increasing evidences connecting ACE functions to the pathogenesis of these acquired diseases, it is suggested that ACE plays a vital role in immune functions. Recent studies with mouse models of bacterial infection and tumor suggest that ACE plays an important role in the immune responses of myeloid cells. Inhibition of ACE suppresses neutrophil immune response to bacterial infection. In contrast, ACE overexpression in myeloid cells strongly induced bacterial and tumor resistance in mice. A detailed biochemical understanding of how ACE activates myeloid cells and which ACE peptide(s) (substrate or product) mediate these effects could lead to the development of novel therapies for boosting immunity against a variety of stimuli, including bacterial infection and tumor.

**This article was specially invited by the editors and represents work by leading researchers**.

## Introduction

The renin-angiotensin system (RAS) is a major regulator for blood pressure, fluid and electrolyte balance, in which sequential action of two enzymes, renin and ACE produce a bioactive peptide angiotensin II (Ang II) [1, 2]. It is well established that RAS, via the Ang II AT1 receptor, plays a crucial role in cardiovascular and renal functions by regulating blood pressure, electrolyte and volume homeostasis [2, 3]. This traditional concept of the RAS as a circulating endocrine system has been evolved enormously with time and many new RAS-regulatory components including peptide molecules, enzymes and G protein-coupled receptor have been identified [4–7]. The development of ACE inhibitors (ACEi, eg. enalapril, captopril, lisinopril, ramipril) or AT1 (Angiotensin II receptor type 1) blockers (ARBs, eg. losartan) not only revolutionized the treatment strategies to treat hypertension, but also provided a tool for the discovery of many unknown functions of ACE components in different physiological and pathological mechanisms.

Increasing number of studies suggest that, in different tissues, a local RAS may operate either systemically or entirely independent to the circulating counterpart, and it may act as a whole or in part to meet the specific needs of the individual tissues via autocrine and/or paracrine pathways [8, 9]. For example, in bone marrow (BM), it affects critical steps of blood cell production, such as hematopoietic niche [10], myelopoiesis [11], and the development of other cellular lineages including lymphocytic [12]. There are also enough studies suggesting local operation of RAS in other organs including cardiac, vascular and renal tissues [13–17].

Renin enzyme is highly specific and extremely limited in its tissue expression, while ACE is relatively nonspecific and widely expressed in different tissues [5]. Other than Ang I, ACE can cleave variety of substrates including bradykinin, substance P, tetrapeptide N-acetyl-seryl-aspartyl-lysyl-proline (AcSDKP), enkephalins, neurotensin and others. Because of this wide tissue distribution and substrate specificity, ACE may affect many diverse biological functions, including renal development, fertility, hematopoiesis and immunity [5, 18, 19]. This review aims to discuss new biological functions of ACE in different aspects of the immune response.

### ACE and immunoinflammatory diseases

Inflammation plays a critical role in immune activation. Many studies have found ACE to be a potent pro-inflammatory modulator [20] that plays a role in the recruitment of inflammatory cells into tissues by regulating chemokines and adhesion molecules [21]. ACE not only functions as a cell membrane ectopeptidase, but it can be secreted into extracellular milieu by activated myelomonocytic or other lineage cells, and thus, it act as both local and systemic regulator of peptides [22]. The association of ACE with immunoinflammatory diseases has been well established. First report published in 1975 that showed higher serum ACE in patients with sarcoidosis. Now, it is known that circulating ACE is elevated in many other granulomatous diseases, such as Gaucher’s disease and tuberculosis [23, 24]. In sarcoidal granulomas the increased ACE activity is predominately contributed by epithelioid cells and macrophages of the granuloma, and a higher serum level of ACE is observed in a majority of patients [25]. In tuberculosis granuloma, increased ACE is mostly contributed by alveolar macrophages [26].

Based on CD14 and CD16 expression, monocytes can be divided into two subpopulations Mo1 and Mo2. Percentage of Mo2 increased in many inflammatory diseases including chronic kidney disease (CDKs), which expresses higher level of ACE along with CD14 and CD16 [17, 27]. These monocytes are proinflammatory in nature and associated with increased atherosclerosis and cardiovascular mortality in hemodialysis patients [28]. It is now believed that all RAS components express in immune cells. For example, monocytes and macrophages express renin, ACE, Ang I and Ang II, AT1 and AT2 receptors [29]. In nervous system also, all RAS components are locally synthesized by different cell types, including astrocytes, microglia and neutrons [30, 31], and ACE expression is increased in many neurological autoimmune diseases including encephalitis and multiple sclerosis [18, 32]. There is no clear understanding why ACE expression increases in these immunoinflammatory conditions. One possibility is that ACE activity increases the activation of immune cells in response to immune challenge.

### ACE in hematopoiesis and myeloproliferation

BM is a complex and highly organized system that produces all circulating cells. However, hematopoiesis is tightly regulated by a variety of factors including enzymes, receptors, hormones, cytokines, growth factors and bioactive peptides. The early clues about the RAS effects on hematopoiesis came from the reduced hematocrit of patients, who were treating with the ACEi enalapril [33]. Studies further suggested that enalapril caused erythrocytosis in patients with renal transplantation by reducing the hematocrit level [34, 35]. In rare patients, ACEi treatment also causes anemia due to its effects on immune system [36]. In clinical studies, people find that ACEi suppress RBC production and decrease white blood cells In clinical studies [37, 38]. These finding clearly hypothesize a possible role of the RAS in hematopoiesis. Over time, all the known components of the RAS, such as angiotensinogen, renin, ACE, AT1a, AT2 (Angiotensin II receptor type 2), Mas (G protein-coupled receptor) and ACE2 (Angiotensin converting enzyme 2), have been identified in the BM, and it is now believed that local RAS operates in the BM [8, 39]. These RAS components, particularly ACE-mediated peptides, affect several critical steps of hematopoietic cell development in physiological and pathological conditions [8, 9, 39].

The existence of ACE in primitive lympho-hematopoietic cells, embryonic and fetal tissues suggests that ACE not only affects erythropoietic progenitors, but might have effects on neoplastic tissues and primitive pluripotential hematopoietic stem cell populations [40, 41]. The ACE substrate Ac-SDKP has been extensively studied in the regulation of stem cell proliferation. ACE inhibition by enalapril increases the level of Ac-SDKP in both plasma and BM [42, 43], which in turn prevents hematopoietic stem cell proliferation [44]. In the BM, stromal cells produce a large amount of Ac-SDKP [43], and the increased ACE expression in stromal cells significantly decreases the Ac-SDKP level in the BM microenvironment. Rousseau-Plasse et al. (1996) demonstrated that ACE plays an important role in the recruitment of primitive stem cells into S-phase by hydrolyzing Ac-SDKP [45]. Comte and colleagues then observed significant changes in the circulatory hematopoietic progenitors in healthy volunteers following administration of enalapril. It increased plasma and urinary Ac-SDKP in association with an increased number of mixed colony-forming unit (mixed-CFU) hematopoietic progenitors, and in contrast, reduced the number of granulocyte-monocytic colony-forming unit (CFU-GM) and Burst-forming unit-erythroid (BFU-E) hematopoietic progenitors in the circulation [46]. Ang II is another important peptide of ACE implicated in hematopoietic cell development. AT1 receptor is expressed by CD34^+^ hematopoietic progenitors and stromal cells in human tissues [47]. By binding to AT1a receptor on CD34^+^ hematopoietic stem cells, Ang II increases proliferation of these cells [47]. Further study validated that ACE inhibition by captopril caused myelosuppression by inhibiting stem cell and progenitor cell proliferation rather than depleting the BM cells [48]. These studies suggest that ACE has a regulatory role in hematopoiesis.

**Fig. 1:**
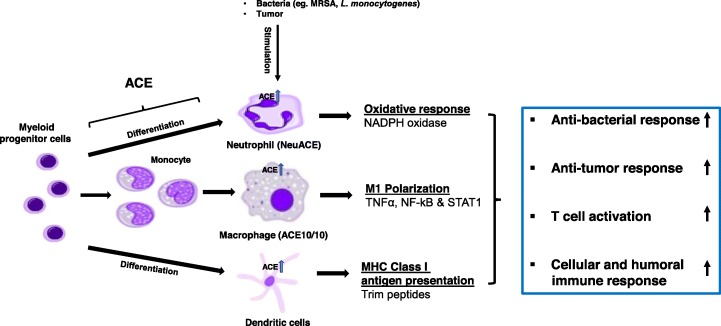
ACE upregulation enhances myeloid cell immune responses. In physiological conditions, ACE expression increased during the differentiation and functional maturation of myeloid-derived cells. Upon immune challenge, the expression of ACE further increased in activated myeloid cells facilitated the optimal immune responses of these cells. Upregulation of ACE in myeloid cells (eg. NeuACE neutrophils and ACE10/10 macrophages) strongly enhanced immune responses of these cells, beyond the normal capacity of WT cells. In neutrophils, ACE upregulation induced oxidative bactericidal response, which is due the upregulation of NADPH oxidase activity. In macrophages, ACE upregulation enhanced M1 activation of macrophages due to the increased activation of NF-kB, STAT1 and TNFα, which in turn gives a strong anti-bacterial and anti-tumor phenotype. In APCs (DCs and macrophages), ACE trims the peptide repertoire before they are bound to MHC class I complex and displayed by cells, which activates T cell – adaptive immune response and humoral immune response

There is now abundant evidence that ACE affect myelopoiesis. These data come from genetically modified mice with either ACE knockout (KO) or selective overexpression of ACE in myeloid cells. Absence of ACE suppresses differentiation of myelomonocytic precursors in ACE KO mice [11]. These mice showed a higher percentage of immature myeloid precursors including myeloblasts, myelocytes, and metamyelocytes as compared to WT mice. In contrast, a significant reduction of mature neutrophils was found in ACE-KO BM as compared to WT BM [11]. A dramatic reduction in nucleated erythroid precursors was found in the BM of ACE KO mice leading to anemia as compared to WT mice. ACE inhibition also increases early BM progenitors, such as LSK cells (a fraction enriched for hematopoietic stem cells) in ACE KO mice BM [11]. In addition, increased extramedullary hematopoiesis was found in the spleen that caused an expansion of immature myeloid cells in ACE KO mice as compared to WT [11]. Similar myelopoiesis abnormalities were observed by pharmacologic inhibition of ACE in mice. In conclusion, these data suggest that ACE knockout enhanced myeloproliferation with reduced differentiation that ultimately increased the number of immature myelomonocytic lineage cells in mice (Fig. 1).

### ACE and myeloid cell immune response

Myeloid cells are a heterogenous population derived from a common myeloid progenitor that belongs to the innate immune system. As a first line of defense, myeloid cells (neutrophils, macrophages, and dendritic cells) are recruited to the inflammatory site and elicit a variety of immune responses to eliminate potential threats from outside and inside. Also, macrophages and dendritic cells (DCs) function as classical antigen presenting cells (APCs), therefore, these cells are crucial for the initiation of an adaptive immune response. There is now strong evidence that ACE facilitates the immune activation of myeloid cells, as discussed below (Fig. 1).

#### Neutrophil immune response

Neutrophils, also called polymorphonuclear leucocytes, are an essential component of the innate immune response which plays a major role during acute inflammation [49]. In blood, mature neutrophils are derived from the BM with the goal to locate and kill harmful invading pathogens [50]. In a lipopolysaccharide (LPS)-induced acute lung inflammation mouse model, when mice were pre-treated with the ACEi enalapril, it reduced pulmonary recruitment of neutrophils following LPS treatment [51]. This may demonstrate that ACE could assist in neutrophil recruitment during pulmonary inflammation.

**Fig. 2:**
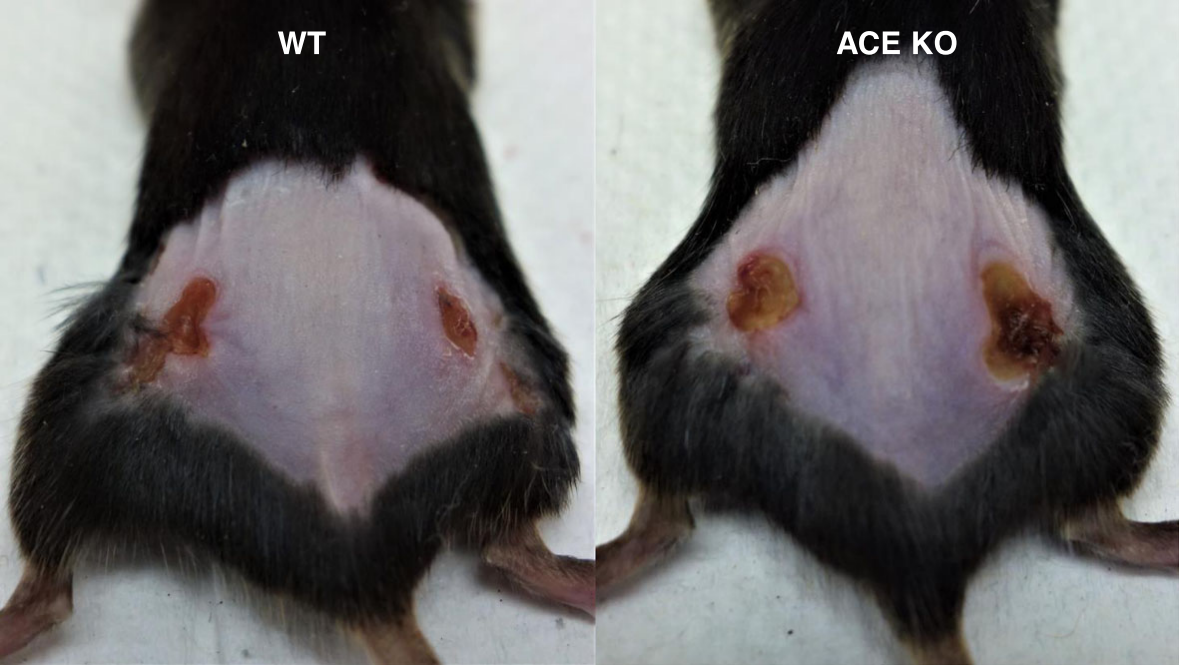
ACE affects anti-bacterial defense. In mice, ACE knockout suppressed bacterial resistance. Representative images showing MRSA skin lesion at day 4 post-bacterial subcutaneous injection (1 × 10^7^ CFU/mouse flank) [Cao D-Y et al. Unpublished data]

As protein expression markedly changes in activated neutrophils, we evaluated the expression of ACE in neutrophils following methicillin-resistant *Staphylococcus aureus* (MRSA) challenge. ACE expression upregulated in neutrophils upon activation with MRSA [52]. To study whether ACE expression is associated with immune functions of neutrophils, ACE KO and WT mice were subcutaneously challenged with MRSA and then mice were sacrificed day 3 post-infection. ACE knockout significantly reduced bacterial resistance in mice as manifested by larger lesion size (Fig. 2) and higher tissue bacterial burden in ACE KO mice as compared to WT mice. To directly access the effect of ACE on neutrophil activity, intracellular bacterial killing by purified ACE KO and WT neutrophils were determined. The intracellular killing of bacteria was significantly higher by WT neutrophils as compared to ACE KO neutrophils [52].

Because the lack of ACE expression is linked with neutrophil immune suppression, we also investigated the phenotype of animals overexpressing ACE in neutrophils. To study this, a transgenic mouse line called NeuACE mice was generated by using a c-*fms*-ACE construct [52]. These mice overexpress ACE (~ 10 fold) in neutrophils. In contrast to ACE KO, NeuACE mice show enhanced resistance to bacterial infections as compared to WT mice [52]. To investigate the role of Ang II, mice were pre-treated with either the losartan (ARB) or ramipril (ACEi) for several days before infection. Ramipril treatment eliminated any differences between NeuACE and WT mice. In contrast, losartan had no major effect [52]. This indicates that AngII-AT1R does not mediate ACE effect on neutrophil antibacterial activity. By using renin inhibitor and Ang II infusion in mice, we confirmed that no Ang peptide mediates neutrophil-ACE effects. Further, the phenotype of ACE overexpressing NeuACE neutrophils appears independent of AcSDKP, bradykinin-B2K receptor and substance P-NK1 receptor [52]. Sometimes, treatment with ACEi caused angioedema (a mucosal immunopathological condition) in patients due to inhibition of kinin degradation and consequent inhibition of kinin-B1KR signaling pathway [53, 54]. However, the role of B1KR in neutrophil immune response needs to be investigated.

The production of reactive oxygen species (ROS) is very important for neutrophil antimicrobial activity. We found that ACE directly affects ROS production in neutrophils. Upon immune challenge, ACE KO neutrophils produce less ROS, while NeuACE neutrophils produce more ROS as compared to WT neutrophils (Fig. 1). Further, our study demonstrated that ACE increases neutrophil antibacterial activity by enhancing NADPH oxidase enzyme activation, an effect that is independent of the angiotensin II AT1 receptor [52].

ACEi are generally considered safe and used by millions of patients for the treatment of hypertension and cardiovascular diseases. As ACE plays an important role in neutrophil anti-bacterial activity, any neutrophilic immune suppression by ACEi may increase risk of infection in vulnerable patients, such as patients with weak immunity. Indeed, some clinical studies have found an association between the uses of ACEi and increased risk of infection including sepsis and urinary tract infection [55–57]. Such infections were not noted with an ARB [57]. Therefore, our findings caution in the use of ACEi under conditions where patients are vulnerable to infections. The uses of ACEi and risk of infection needs to be further investigated.

#### Macrophages immune response

Macrophages as major residential innate cells can be found in many tissues and organs. They play a variety of roles in innate immune responses, such as phagocytosis, cytokine secretion, and antigen presentation etc. As mentioned above, increased ACE expression in granulomas was predominately contributed by epithelioid cells and macrophages. Also, ACE was upregulated during monocyte differentiation into macrophages in both the THP-1 cell line cultured with adipocyte-derived lipids [58] and in human peripheral blood [59, 60]. Under uremic conditions, up-regulation of ACE in primary monocytes and THP-1 cells enhanced differentiation of these cells to macrophages with induced expression of proinflammatory cytokines, adhesion and transmigration molecules [61]. In mice, ACE upregulation was found in macrophages and DCs following MRSA or *Listeria monocytogenes (L. monocytogenes)* infection [62, 63] (Fig. 1). These findings show link between ACE upregulation and the activation of myeloid cells. What remained unknown was whether ACE upregulation also contributed in enhancing immune responses of these cells. To study this, Dr. Shen and colleagues developed a recombinant mice line, called ACE10/10 that overexpressed ACE in macrophages [62].

##### Anti-bacterial response 

To study macrophage innate immune response, the resistance of the ACE10/10 mice to MRSA or *L. monocytogenes* was determined [63]. For MRSA infection, mice were challenged subcutaneously, and then at day 4, mice were sacrificed and skin lesion size and lesional bacterial number were determined. ACE10/10 mice showed strong resistance to MRSA infection, as manifested by smaller lesion size and significantly lower lesional bacteria count as compared to WT mice. ACE10/10 mice also showed resistance to *L. monocytogenes*; a significantly lower tissue bacteria count (spleen and liver) was found in ACE 10/10 mice as compared to WT mice [63]. In addition, a reduction of necrosis and abscess formation in ACE10/10 mice further validated bacterial resistance in these animals as compared to WT mice. These differences between ACE10/10 and WT mice were not due to the Ang II AT1 receptor, as there was no effect of losartan treatment [63]. Furthermore, in vitro study with peritoneal macrophages showed that ACE does not participate directly in bacterial killing because without priming with interferon-gamma (IFNγ), no difference was found between ACE10/10 and WT macrophages [63]. Nitric oxide (NO) is critical for a macrophage anti-microbial response to *L. monocytogenes and* MRSA. It was found that ACE upregulation increased NO production in macrophages leading to an improved immune response to bacterial infection [63].

##### Anti-tumor response

Macrophages are not only important for the initial innate immune response but also play a crucial role in the initiation of the adaptive immune response by functioning as APCs and activating T cells. Shen et al. (2007) investigated macrophage anti-tumor response in relation to ACE expression [62]. They found that ACE overexpression in macrophages increased tumor resistance in mice. B16-F10 melanoma growth was significantly lower in ACE10/10 mice as compared to WT mice (Fig. 1). This tumor resistance in ACE 10/10 mice was dependent on increased number of tumor epitope-specific CD8^+^ T cells, as depletion of CD8^+^ T cells led to rapid tumor growth in ACE 10/10 mice [62].

**Fig. 3:**
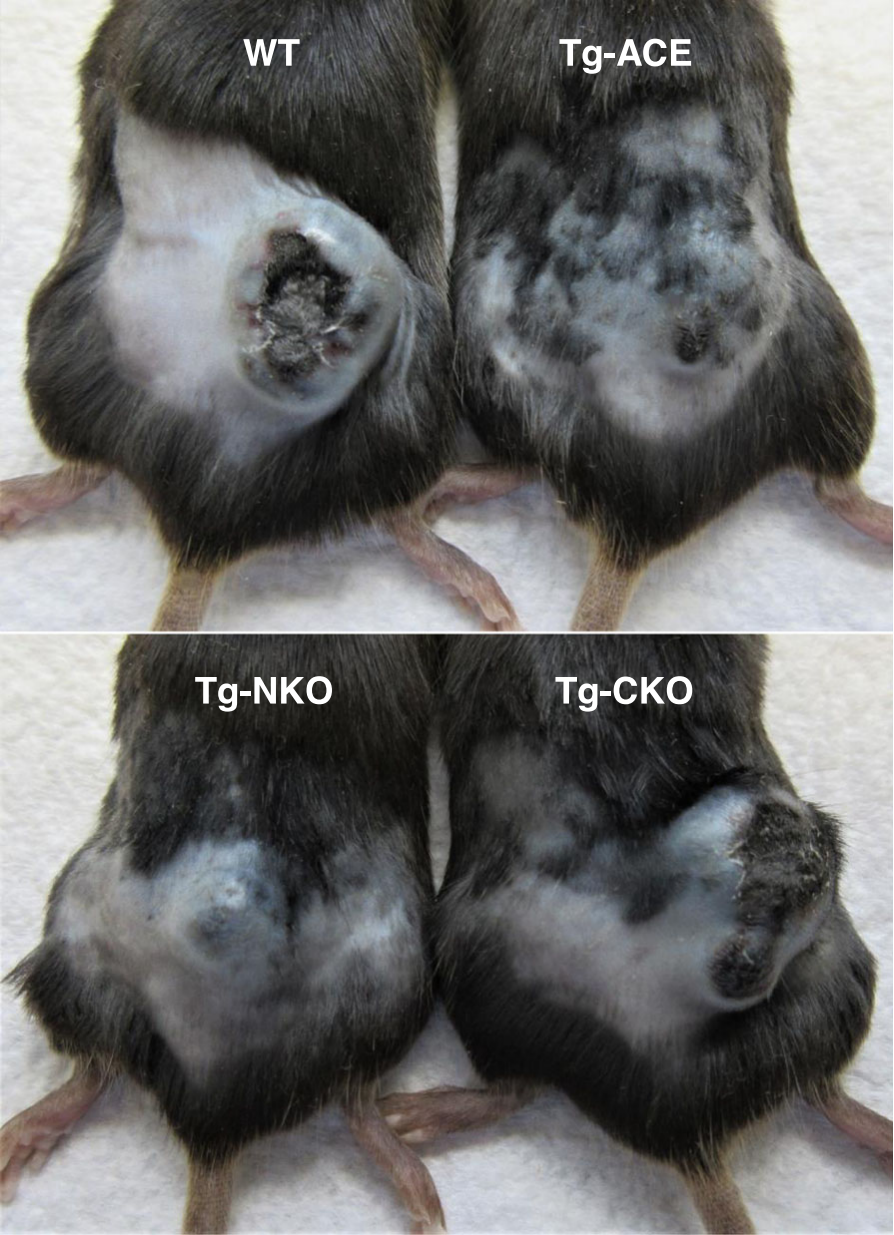
Melanoma tumor growth. In mice, overexpression of ACE C-domain enhanced macrophage anti-tumor activity. Representative images showing tumor growth at day 14 after intradermal injection of B16-F10 melanoma cells (10^6^ cells/mouse) [Cao D-Y et al. Unpublished data]

ACE consists of two independent catalytic domains (N- and C-domains) [5]. To study the specific role of each domain, transgenic mice were generated, which overexpressed either WT ACE (Tg-ACE mice) or ACE lacking N- or C-domain catalytic activity (Tg-NKO and Tg-CKO mice) in myeloid cells. Tg-ACE and Tg-NKO mice strongly suppressed the growth of melanoma. In contrast, Tg-CKO mice resist melanoma no better than WT mice (Fig. 3) [64]. Thus, this study shows that the overexpression of ACE C-domain by macrophages is a strong mechanism to increase resistance to tumor. Ang II is a very important peptide generated by the C-domain of ACE [5]. To study whether the Ang II-AT1 axis mediates tumor resistance, tumor growth was determined in mice pre-treated with losartan before tumor implantation. However, we found no significant effect of losartan on tumor growth suggesting this pathway does not participate in ACE mediated tumor resistance in mice. Further, blocking other known ACE C-domain peptide pathways, such as bradykinin-B2R and substance P-NK1R, had no effect on tumor growth in mice [64]

.

It is found that up-regulation of ACE C-domain induced M1 signals in macrophages, including activation of tumor necrosis factor alpha (TNFα), nuclear factor kappa-light-chain-enhancer of activated B cells (NF-κB), and signal transducer and activator of transcription 1 (STAT1), and in contrast inhibition M2 signals including STAT3 and STAT6 was found. This appears to reprogram these cells towards a more classically activated M1 phenotype that is responsible for the enhanced tumor resistance (Fig. 1) [64].

##### Metabolic effects

Metabolic reprogramming is closely associated with the polarization of macrophages [65]. For example, LPS dependent inflammation by M1 macrophages mostly relies on glycolysis and fatty acid biosynthesis. In contrast, tissue repair activity by M2 macrophages switches their metabolism to fatty acid oxidation and oxidative phosphorylation [65]. To investigate the molecular basis of how ACE affects cells phenotypically, metabolism of ACE overexpressing myeloid cells (ACE10/10 and NeuACE) was determined. It was found that ACE up-regulation significantly increased ATP, Krebs cycle intermediates and electron transport chain proteins (NDUFB8, ATP5A, and ATP5β) in ACE10/10 macrophages and NeuACE neutrophils as compared to WT cells [66]. This appears to underpin some of the phenotypic differences between these cells and myeloid cells expressing WT levels of ACE, such as superoxide production and phagocytic bacterial killing. Consistent with anti-tumor response, the C-domain of ACE predominately enhanced ATP production in these cells. Again, there was no effect of Ang II on myeloid cell ATP production [66].

#### Dendritic cells and adaptive immune response

ACE is also expressed by dendritic cells (DCs) [67, 68]. Ang II and bradykinin have been implicated in DCs maturation and Th1 cell development in a mouse model of *Trypanosoma cruzi* infection [69]. ACE expression increased during the differentiation of DCs and was further increased when these cells were activated with the pro-inflammatory cytokine IFN-γ [70]. Similarly, when mice were challenged with listeria, ACE expression was up in splenic macrophages and DCs [70]. Major histocompatibility complex (MHC) class I proteins play a critical role in the activation of CD8^+^ T cell adaptive immune responses against intracellular pathogens and perhaps also against tumors. APCs, such as DCs and macrophages phagocytize cellular debris and present MHC class I bound antigens for CD8+ T cells. Mice studies suggest that ACE can trim the peptide repertoire displayed on the surface of APCs as part of the MHC class I complex (Fig. 1) [70, 71].

In addition to myeloid cells, ACE also plays a role in the activation of lymphoid cells. Hoch et al. (2009) found a direct autocrine effect of ACE and Ang II on T cell function, including activation, expression of tissue-homing markers, and production of cytokines [72], probably due to superoxide production by T cell NADPH oxidase. In a rodent model of cerebral malaria, following activation by infection, T cells show a significant increase of CD69 expression, while this reduced to normal when mice were treated with losartan and captopril [73]. In a clinical study of pulmonary sarcoidosis, the percentage of lymphocytes and the CD4/CD8 ratio in bronchoalveolar lavage fluid (BALF) is coordinated with ACE activity [74]. Further, a connection between Th1 cell cytokines (IL-12 and IL-18) and ACE activity was determined in BALF [75]. ACE and Ang II have also been implicated in encephalitis [17]. All these findings suggest an active participation of ACE in adaptive immune activation. Unfortunately, the critical ACE function and catalytic substrates are still obscure and need more investigation.

## Conclusions

ACE is one of the most vital and well-studied peptidases in the RAS. However, in recent years, ACE functions are found to coordinate with immune responses. ACE is upregulated in many immunological diseases, such as granuloma. Similarly, upregulation of ACE was reported in myeloid cells following immune challenges. By comparing ACE KO, WT and ACE overexpressing neutrophils and macrophages, our studies have demonstrated that ACE not only plays a physiological role in myeloid cell immune response, but if overexpressed, ACE further enhances immune responses against a variety of stimuli, such as bacterial infection and tumor, which is beyond the normal ability of WT cells. However, whether ACE overexpression has similar effects on human myeloid cells needs to be investigated. In both tumor and infection studies with transgenic mice overexpressing only an active C-domain or an active N-domain, we found that the C-domain of ACE is important for increasing the immune responses of myeloid cells. However, none of the known ACE C-domain peptides, such as all angiotensin peptides, bradykinin and Substance p were found to mediate these ACE effects on neutrophils and macrophages. Identification of ACE peptide(s) (substrate or product) that elicit an increased immune response may hold great promise for therapeutic manipulation to boost the immune response against a variety of stimuli, including infections and tumors.

## Abbreviations

ACE: Angiotensin-converting enzyme
RAS: Renin-Angiotensin System
Ang II: Angiotensin II
ACEi: ACE inhibitors
ARBs: AT1 blockers
BM: Bone Marrow
AcSDKP: Tetrapeptide N-Acetyl-Seryl-Aspartyl-Lysyl-Proline
WT: Wild type
ACE KO: ACE Knockout
NeuACE: Neutrophil ACE overexpression
Tg-ACE: Transgenic ACE
Tg-CKO: Transgenic ACE C domain knockout
Tg-NKO: Transgenic ACE N domain knockout
APCs: Antigen Presenting Cells
DCs: Dendritic Cells
LPS: Lipopolysaccharide
MRSA: Methicillin-Resistant *Staphylococcus aureus*
*L. monocytogenes*: *Listeria monocytogenes*
IFNγ: Interferon-gamma
NO: Nitric oxide
TNFα: Tumor Necrosis Factor alpha
NF-κB: Nuclear Factor Kappa-light-chain-enhancer of activated B cells
STAT: Signal Transducer and Activator of Transcription
MHC: Major Histocompatibility Complex
BALF: Bronchoalveolar Lavage Fluid
